# Do monkey F5 mirror neurons show changes in firing rate during repeated observation of natural actions?

**DOI:** 10.1152/jn.01102.2012

**Published:** 2013-12-26

**Authors:** J. M. Kilner, A. Kraskov, R. N. Lemon

**Affiliations:** ^1^The Wellcome Trust Centre for Neuroimaging, Univeristy College of London Institute of Neurology, London, United Kingdom; and; ^2^Sobell Department of Motor Neuroscience and Movement Disorders, Univeristy College of London Institute of Neurology, London, United Kingdom

**Keywords:** action observation, F5, mirror neurons, repetition suppression

## Abstract

Mirror neurons were first discovered in area F5 of macaque monkeys. In humans, noninvasive studies have demonstrated an increased blood oxygen level-dependent (BOLD) signal in homologous motor areas during action observation. One approach to demonstrating that this indicates the existence of mirror neurons in humans has been to employ functional (f)MRI adaptation to test whether the same population of neurons is active during both observation and execution conditions. Although a number of human studies have reported fMRI adaptation in these areas, a recent study has shown that macaque mirror neurons do not attenuate their firing rate with two repetitions. Here we investigated whether mirror neurons modulate their firing rate when monkeys observed the same repeated natural action multiple times. We recorded from 67 mirror neurons in area F5 of two macaque monkeys while they observed an experimenter perform a reach-to-grasp action on a small food reward using a precision grip. Although no changes were detectable for the first two repetitions, we show that both the firing rate and the latency at which mirror neurons discharged during observation were subtly modulated by the repetition of the observed action over 7–10 trials. Significant adaption was mostly found in the period immediately before the grasp was performed. We also found that the local field potential activity in F5 (beta-frequency range, 16–23 Hz), which is attenuated during action observation, also showed systematic changes with repeated observation. These LFP changes occurred well in advance of the mirror neuron adaptation. We conclude that macaque mirror neurons can show intra-modal adaptation, but whether this is related to fMRI adaptation of the BOLD signal requires further investigation.

mirror neurons are a class of neuron that was first discovered in the ventral premotor area F5 of the macaque monkey (di Pellegrino et al. 1992; Gallese et al. 1996; Umilta et al. 2001; Rizzolatti et al. 2010) and subsequently demonstrated to be also present in a region of the inferior parietal lobule, area PF (Gallese et al. 2002; Fogassi et al. 2005). The original defining property of mirror neurons is that they discharge not only when the monkey executes a certain action but also when the monkey observes a similar action performed by an experimenter. Human neuroimaging studies have provided evidence of activation in homologous cortical areas when humans observe and execute actions (Gazzola and Keysers 2009; Buccino et al. 2001; Decety et al. 1997; Grezes and Decety 2001; Hamilton and Grafton 2006; Rizzolatti et al. 1996). In addition, functional (f)MRI studies employing the repetition suppression technique have shown significant fMRI adaptation when actions are repeatedly observed (Dinstein et al. 2007; Chong et al. 2009; Lignau et al. 2009 Kilner et al. 2009; Press et al. 2012). Some authors have interpreted this as being consistent with the presence of mirror neurons in the human inferior frontal gyrus. However, the fMRI results are difficult to interpret in this way as the only study to date that has directly addressed the question of whether or not repetition suppression can be demonstrated in the responses of single mirror neurons has failed to find any evidence of repetition effects in F5 mirror neurons (Caggiano et al. 2013).

There are three underlying neural mechanisms that have been proposed to explain neuronal adaptation with repetition (cf. Grill-Spector et al. 2006). These are the “fatigue model,” where the decrease in the neuronal response is caused by a decrease in the firing rate of each neuron with repetition; the “sharpening model,” where fewer neurons discharge with repetition; and finally, the “facilitation model,” where the decrease in the neuronal response reflects changes in the synaptic potentiation between neurons, enabling a faster and more efficient processing of the stimulus leading to a decrease in the latency, duration, and firing rate of each neuron's discharge with each repetition. Whereas the fatigue model and sharpening models are proposed to be driven by bottom-up stimulus specific features, the facilitation model is thought to be driven by top-down modulations in the expectation of the forthcoming stimulus (Friston 2005). Indeed recent studies have shown that some repetition suppression effects can be explained by modulations in stimulus expectation (Summerfield et al. 2008).

The aim of the analysis presented here was to investigate whether there were any modulations in the firing pattern of mirror neurons with repeated observations of the same natural action that could explain the reported fMRI adaptation effects. To this end we analyzed the effect of stimulus repetition on the activity of macaque mirror neurons and of local field potentials (LFPs) recorded from area F5 when monkeys observed an experimenter repeatedly performing a reach-to-grasp action. We show, in agreement with Caggiano et al. (2013), that there is no clear modulation in firing rate of mirror neurons over two presentations. However, we show that both the firing rate and the latency at which mirror neurons discharged during action observation are subtly modulated by multiple repetitions of the same action. Discharge onset latency and firing rate were both reduced with successive repetitions. In addition we show that the power of LFP oscillatory activity in the beta-frequency range is modulated with repetition of the observation of an action.

## METHODS

Single neuron recordings were made from two purpose-bred adult macaque monkeys, as described previously (Kraskov et al. 2009). All experimental procedures were approved by the Local Ethical Procedures Committee and carried out in accordance with the UK Animals (Scientific Procedures) Act.

**Fig. 1.**
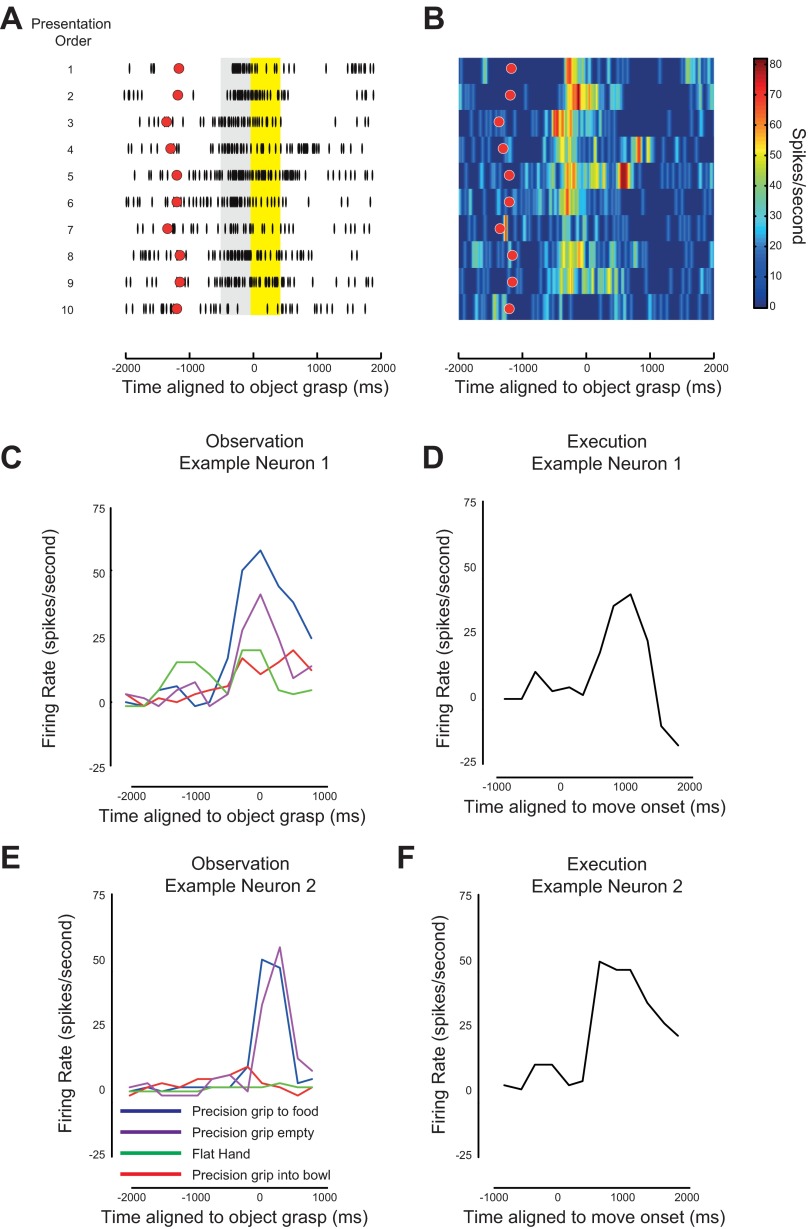
Mirror neuron showing adaptation during observation of a repeated grasp. *A*: firing pattern of the same representative F5 mirror neuron in 10 successive trials (vertical black dashes) aligned to the moment the object was grasped (time zero). Trial number increases from *top* to *bottom*. Red dots show the relative time of the onset of the observed action. The gray box indicates the 500 ms before the observed grasp and the yellow box indicates the 500 ms after the observed grasp. *B*: same data as in *A* but plotting the firing rate of the neuron as a function of perievent time (object grasp) and presentation order. As before the red dots show the relative onset of the observed action. Note the systematic decrease in firing rate with successive trials. *C* and *E*: examples of 2 mirror neurons during observation of different actions. Blue lines show the response when the monkey observed a precision grip to pick up a piece of food, red lines show the response of the neuron when observing a precision grip to a piece of food concealed in a bowl, purple lines show the response when observing a precision grip to no object, and green lines show the response to observing the experimenter placing his/her hand flat in front of the monkey. *D* and *F*: response of these two neurons when the monkey executed a precision grip task.

The action observation procedure comprised blocks of 10 precision grip trials carried out on the table in front of the monkey. Each trial began with the experimenter's right hand resting on a central homepad. About 1 s later a tone sounded that cued the experimenter to release the homepad and begin the reach-to-grasp action towards a small piece of food that was placed on a central sensor (Fig. 1, *A* and *B*, red circles). The experimenter wore a glove on the right hand, and this glove contained a small magnet at the tip of the finger. As the experimenter reached the “action observation area” of the table, which was in the monkey's midline and just beyond its reach, a magnetic sensor embedded in the table at the center of this area was activated and generated a sensor pulse, indicating the onset of the grasp (time zero in Fig. 1, *A* and *B*). The timing of the homepad signal and of this sensor pulse were recorded along with neuronal data (Kraskov et al. 2009). Tests were repeated once every 4–5 s. On average, the monkey was rewarded after every fifth trial. The reward was presented no earlier than 1,000 ms after the observed action was completed. All conditions were tested in blocked trials, and no randomized presentations were used with these monkeys.

### 

#### Initial selection of neurons.

A total of 116 neurons were recorded in F5, of which 64 were physiologically identified as pyramidal tract neurons. Thus most of the neurons in our sample were selected for recording based on their antidromic responses, rather than on their activity during the behavioral task. The otherwise unidentified neurons were recorded simultaneously with the pyramidal tract neurons on other nearby electrodes.

In our initial analysis of the 116 F5 neurons, the following criteria were used to select mirror neurons suitable for studying the possible effects of repetition suppression. First, we included only those neurons that modulated their firing rate both during grasp of a small food reward by the monkey and during observation of precision grip of a small food reward by the experimenter. Second, we included those neurons whose firing rate was modulated during action observation of precision grip, where its mean firing rate in either a 500-ms window before the object grasp (Fig. 1*A*, gray box) or in a 500-ms window after the object was grasped (yellow box Fig. 1*A*) differed from the mean firing rate during a background period, defined as a 500-ms window before the initiation of the observed action, by more than one standard deviation. Third, only data for the first 10 repeats of the action were included in the analyses. Fourth, to ensure that any effects we observed could not be explained by a change in the duration of the observed movement we eliminated trials where the movement duration was <750 ms and >2,000 ms. Any neuron was excluded from further analysis if there were <7 repeats of the observed action remaining.

Based on these four criteria, 49/116 neurons were excluded, leaving 67 candidate mirror neurons that were analyzed further. Two examples of mirror neurons that met these requirements are shown in Fig. 1, *C*–*F*. Note that both neurons increased their firing rate during observation (Fig. 1, *C* and *E*, blue lines) and execution (Fig. 1, *D* and *F*) of a precision grip. In addition, both these neurons showed differing levels of firing rate response when observing different actions (see Kraskov et al. 2009). We do not exclude the possibility that these neurons may have also responded to other actions (execution/observation); however, the difference in firing rates to the different actions tested demonstrate that these neurons were not simple visuomotor neurons, discharging equally to any action.

To produce a smooth estimate of the firing rate, the spike train data were convolved with a Gaussian with a full-width half maximum of 100 ms that was normalized so that it summed to 1. To investigate changes in firing rate with trial with respect to time, the spike train data were divided into 250-ms nonoverlapping bins and the number of spikes in each bin was calculated.

To calculate whether there was a systematic change in firing rate with repeated presentation we performed two different analyses. Firstly, for each neuron the number of spikes in each 250-ms bin was correlated with repetition number. This was performed using code written in Matlab (Mathworks). For each time bin and each neuron we obtained the beta value from a linear regression analysis where the model was the repetition number and a constant term. The sign of the first beta coefficient indicated the direction of modulation of firing rate with repetition. A negative value would indicate a decrease in firing rate where as a positive value would indicate an increase. All population level statistics were performed on these beta values. It is important to note that the regression on the individual neurons was employed to calculate the beta values. The population level statistics do not require that the regression be significant at the individual neuron level. This analysis was performed for data aligned to time of object grasp and for data aligned to time of movement onset. Secondly, we calculated the adaptation index (AI) for each repetition, which was defined as the difference in firing rate between different number of repetitions of the same action. These were averaged across neurons to produce a population level AI, analogous to activity in one voxel in fMRI measures. In this way an increasing value of AI with repetition would indicate a decrease in firing rate with repetition.

To identify any change in the onset of firing rate change we performed the following analysis for each neuron. For each trial the time of the “center of mass” of spikes in a 1,000-ms window of interest centered on the time of the peak of the average firing was calculated. To this end each time point in the 1,000-ms window was weighted by whether there was a spike at that time point, and this was then averaged and normalized by the total number of spikes in the 1,000-ms window. This measure was then correlated with repetition number to determine if there was any systematic modulation in latency with repetition. To exclude the potential confound that this latency measure was modulated by the time the reach was initiated, the time of trial start was included in the regression. In this way, for each neuron, we obtained the beta value from the regression analysis indicating the direction of modulation in the onset latency with repetition. A negative value would indicate a decrease in the latency of firing rate, whereas a positive value would indicate an increase. All population level statistics were performed on these beta values. This measure of center of mass of spikes is very sensitive to trials in which there was little or no discharge. We therefore excluded any trials in which the neuron did not discharge more than four spikes in the 1,000-ms window of interest. Of the sample of 67 mirror neurons selected, 51 met the criterion for this part of the analysis.

#### Analysis of LFP data.

LFPs were recorded from 64 sites in F5 through the same microelectrodes used for spike recording. The LFP was analyzed in the frequency domain. The LFP time series was aligned either to the time of object grasp or the time of movement onset, and a time-frequency map of the LFP data was produced by a fast Fourier transform of the time series. The log of the resulting power spectra were taken. In an initial analysis, the power spectra for each recording were averaged over trials and the power in a 500-ms window before movement onset was subtracted for each frequency bin. The resultant time-frequency maps were then analyzed in SPM8 (Kilner et al. 2005; Kilner and Friston 2010). First, the time-frequency maps were smoothed with a 2-D Gaussian kernel with a full-width half maximum of 112 ms in the time dimension and 1.95 Hz in the frequency dimension. The resultant smoothed time-frequency images were then tested to see if any pixel in the image was significantly greater or smaller than zero. All statistics are reported at a family-wise error (FWE) corrected *P* value of 0.05 using random field theory (Kilner et al. 2005; Kilner and Friston 2010). All subsequent analysis focused on modulation in the power in the beta-frequency range identified from the previous analysis as a frequency window from 15 to 23 Hz. To test if there was a systematic modulation in beta power with repetition, the power was averaged across the 15- to 23-Hz band and then correlated with presentation order for each time point and each electrode recording independently. In this way, for each recording, we obtained the beta value from the regression analysis indicating the direction of modulation of beta power with repetition. A negative value would indicate a decrease in beta power, whereas a positive value would indicate an increase. All population level statistics were performed on these beta values.

## RESULTS

### 

#### Characterization of mirror neurons.

**Fig. 2.**
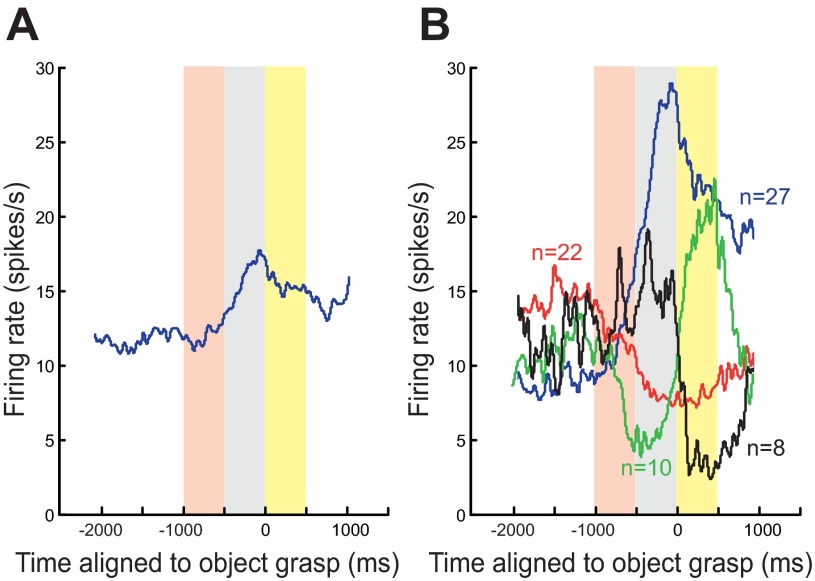
Different patterns of mirror neuron discharge during action observation. *A*: averaged firing rate of all 67 mirror neurons. The pink box indicates the 500-ms window starting 1,000 ms before object grasp (time zero) and was on average the first 500 ms of the observed movement. The gray box indicates the 500 ms before the observed grasp, and the yellow box indicates the 500 ms after the observed grasp. *B*: averaged firing rate for 4 different classes of mirror neurons. Neurons that increased their firing rate during both periods of action observation are shown in blue (*n* = 27), neurons that decreased their firing rate during both periods of action observation are shown in red (*n* = 22), neurons that increased their firing rate before the grasp and decreased their firing rate after the grasp are shown in black (*n* = 8), and neurons that decreased their firing rate before the grasp and increased their firing rate after the grasp are shown in green (*n* = 10).

Of the original 116 neurons, 67 mirror neurons met the requirements for further analysis (see methods). Many of these neurons (42/67) were identified as pyramidal tract neurons (Kraskov et al. 2009). The modulation of the firing rate during action observation of precision grip when aligned to the time at which the object was grasped by the experimenter and averaged across all 67 mirror neurons showed an increase in the firing rate during the period of action observation (Fig. 2*A*, gray and yellow boxes) compared with a baseline premovement period (see Figs. 1 and 2*A*). This population change in firing rate concealed some rather different patterns of modulation, which on closer inspection could be classified into one of four classes. Twenty-seven mirror neurons showed a significant increase in their firing rate during both the period of action observation before the object grasp (Fig. 2, gray boxes) and during the period of action observation after the object was grasped (Fig. 2, yellow boxes) compared with the premovement baseline period (Fig. 2*B*, blue line). We refer to these neurons as “facilitation” mirror neurons. Twenty-two mirror neurons showed a significant decrease in their firing rate during both periods of action observation compared with the premovement period (Fig. 2*B*, red line; we refer to these as “suppression” mirror neurons). Ten mirror neurons showed a decrease in the firing rate during the period of action observation before the grasp and an increase in the firing rate during the period of action observation after the object had been grasped (Fig. 2*B*, green line). Finally, eight mirror neurons showed an increase in their firing rate during the period of action observation before the grasp and a decrease in the firing rate during the period of action observation after the object had been grasped (Fig. 2*B*, black line).

#### Comparison of firing rate from first to second stimulus presentations.

**Fig. 3.**
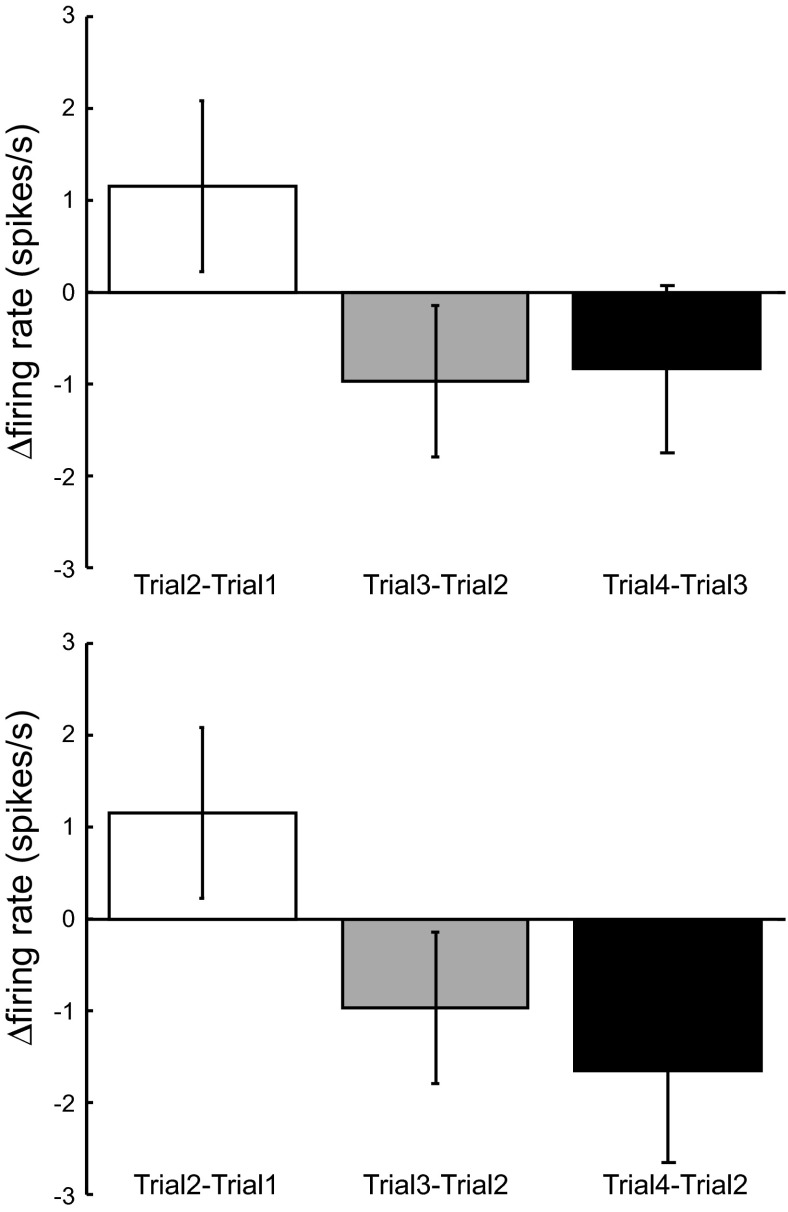
Trial-to-trial modulation in mirror neuron firing rate. *A*: change in mean change in firing rate from *trial 1* to *trial 2* (white bars) *trial 2* to *trial 3* (gray bars) and *trial 3* to *trial 4* (black bars). *B*: same data as in *A* where now the black bars show the difference between *trial 2* and *trial 4*. All error bars at the means ± SE. Data shown for 67 mirror neurons.

An initial analysis investigated whether there was a significant change in the firing rate of mirror neurons from the first to the second observation of the same action. To this end, the average firing rate for each neuron during the period of action observation, a 1,500-ms window starting 1,000 ms before when the object was grasped, was calculated for each repetition, and the difference between the firing rates was calculated, the AI. There was no significant difference in the firing rate between the first and second observation of the same action [*t*(63) = −1.27; *P* = 0.21; Fig. 3*A*, white bars]. Rather than being suppressed, the average firing rate showed a trend to increase from the first to the second presentation. This pattern was observed irrespective of whether mirror neuron firing rate was increased or suppressed during action observation. To investigate this further we calculated the AI for the second to third presentations (Fig. 3*A*, gray bars) and the third to the fourth presentations (Fig. 3*A*, black bars). As before at the population level, there were no significant modulations in the firing rate with subsequent presentations [*t*(58) = 1.12; *P* = 0.27 comparing *trial 2* to *trial 3* and *t*(53) = 0.83; *P* = 0.41 comparing *trial 3* to *trial 4*]. However, with each subsequent presentation there was a trend for the overall firing rate to decrease after an initial trend to increase. This is shown more clearly in Fig. 3*B*.

#### Comparison of firing rate from first to all subsequent presentations.

**Fig. 4.**
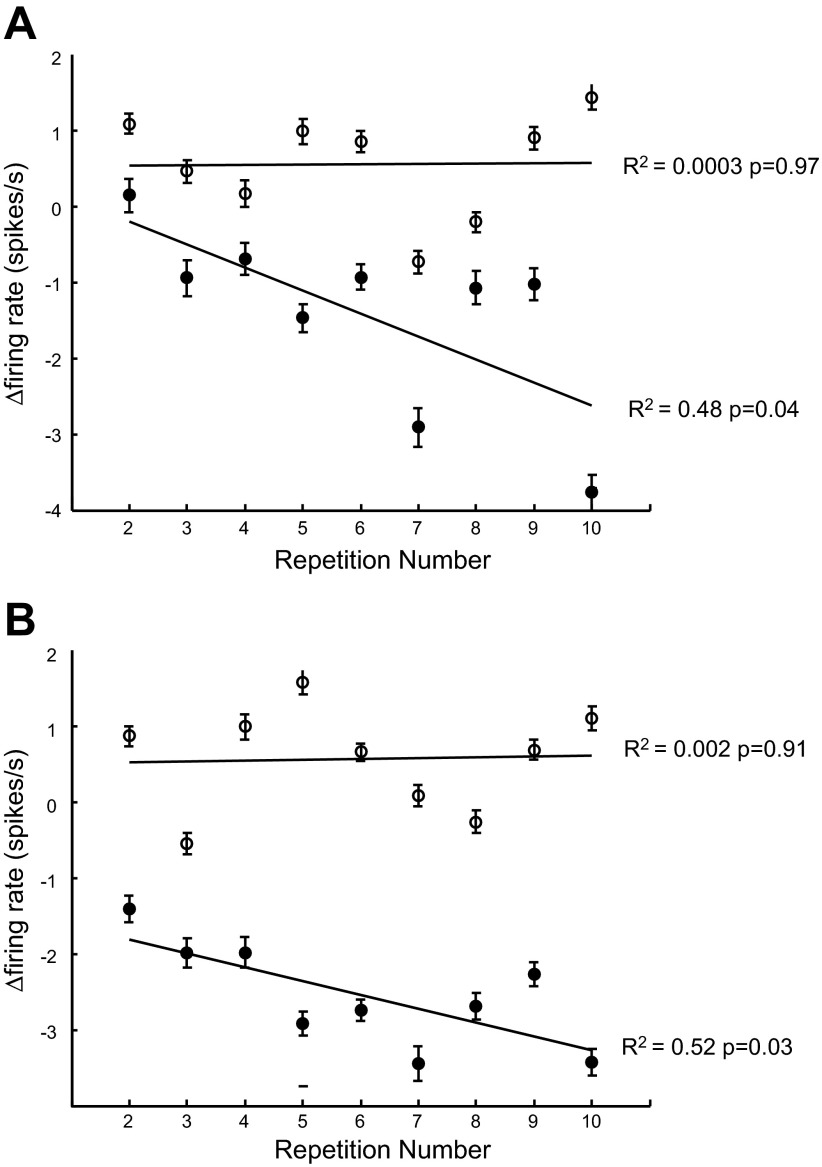
Adaptation of firing rate of mirror neurons with repeated presentations. *A* and *B*: change in firing rate from the first presentation in two 500-ms time windows before object grasp. An increasing value of the adaptation index (AI; see methods) indicates a decrease in firing rate with repetition; note the inverted scale. *A*: AI when the data were aligned to the time of object grasp. *B*: same analysis for data aligned to the start of the observed movement. In *A* the filled circles show the mean AI across neurons for the 500-ms time window before the object grasp. Open circles show the mean AI across neurons for the 500-ms window before the time window used for the filled circles. This window is shown in pink in Fig. 2. In *B* the filled circles show the mean AI across neurons for the 500 ms time window starting 500 ms after the movement began. Open circles show the mean AI across neurons for the 500-ms window just after movement onset. Error bars show SE. The line of best fit from a linear regression analysis is shown for all data. Data shown for 67 mirror neurons.

To investigate the change in firing rate with repetition further, we recalculated the AI for each neuron and averaged these across neurons for two 500-ms windows. When the spike trains were aligned to object grasp, the windows were *1*) the 500 ms before the grasp (Fig. 4*A*, filled circles, and *2*) the 500 ms before that (Fig. 4*A*, open circles). When the spike trains were aligned to the start of the observed movement the windows were *1*) the 500 ms just after movement start (Fig. 4*B*, open circles) and *2*) the 500 ms subsequent to that (Fig. 4*B*, filled circles). For both alignments there was a significant decrease in the AI with repetition in the 500-ms window just before the time when the object was grasped (filled circles), which corresponds to a firing rate decrease. In contrast there was no significant modulation in AI with repetition in the 500-ms window before this (Fig. 4, *A* and *B*, open circles). Indeed, these effects were unaffected by the selection criteria and remained statistically significant when all trials and all neurons were included in the analysis. In light of these results, all subsequent analyses focused on the task-dependent modulations in mirror neuron firing rate across multiple repetitions.

#### Modulation of mirror neuron firing rate with repetition: spike data aligned to grasp.

**Fig. 5.**
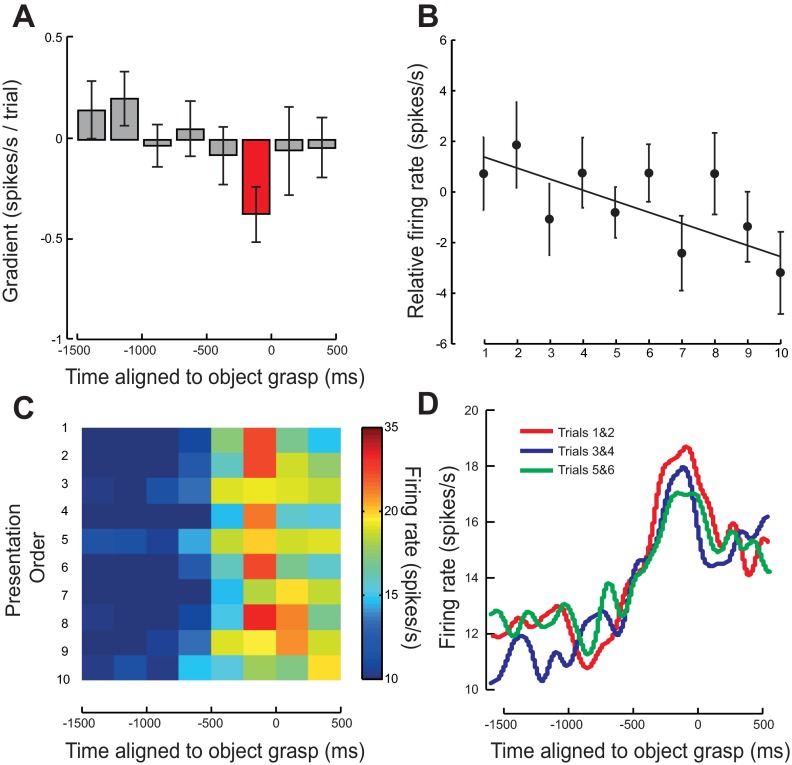
Modulation in mirror neuron firing rate when aligned to the time of object grasp. *A*: average rate of change of firing rate with repetition over 7–10 trials. A negative value indicates a firing rate decrease with repetition. Error bars show means ± SE. Time points where the data were significant are shown in red. *B*: average relative change in firing rate, calculated from the mean firing rate averaged across mirror neurons for the 7–10 presentations of the observed action averaged for the 250 ms before the object being grasped. Error bars show means ± SE. *C*: average firing rate of all mirror neurons for each presentation. Presentation order is shown on the *y*-axis with repetitions increasing from *top* to *bottom*. *D*: average smoothed firing rate across all mirror neuron and averaged across *presentations 1* and *2* (red line), *presentations 3* and *4* (blue line), and *presentations 5* and *6* (green line).

The rate of change of firing rate with presentation for each of the 67 mirror neurons aligned to the time at which the object was grasped showed a systematic and significant decrease in the 250 ms before the object grasp [*t*(66) = −2.69; *P* < 0.01; Fig. 5*A*, red bar]. This decrease in firing rate, 0.48 spikes·s^−1^·trial^−1^, equated to a decrease of ∼5 spikes/s over the 10 presentations of the same action (Fig. 5, *B*–*D*). Note that all significant linear decreases in firing rate with presentation occurred in the period before the object being grasped (Fig. 5, *A*, *C*, and *D*). The average time of movement onset was 1,090 ms (SD 203 ms) before the object being grasped. This same pattern of modulation was observed irrespective of whether the mirror neurons increased or suppressed their firing rate either before the object grasp [facilitation mirror neurons, *t*(30) = −1.92, *P* = 0.06; suppression mirror neurons, *t*(27) = −1.41, *P* = 0.17] or subsequent to the object grasp [facilitation mirror neurons, *t*(34) = −2.00, *P* = 0.05; suppression mirror neurons, *t*(27) = −1.87, *P* = 0.07]. Indeed, this effect was nonsignificant if the sign of the modulation of the suppression mirror neurons was reversed before statistical testing [cf. Caggiano et al. 2013; *t*(66) = 0.18, *P* > 0.05 and *t*(66) = 0.11, *P* > 0.05, respectively].

#### Modulation of mirror neuron firing rate with repetition: data aligned to movement onset.

**Fig. 6.**
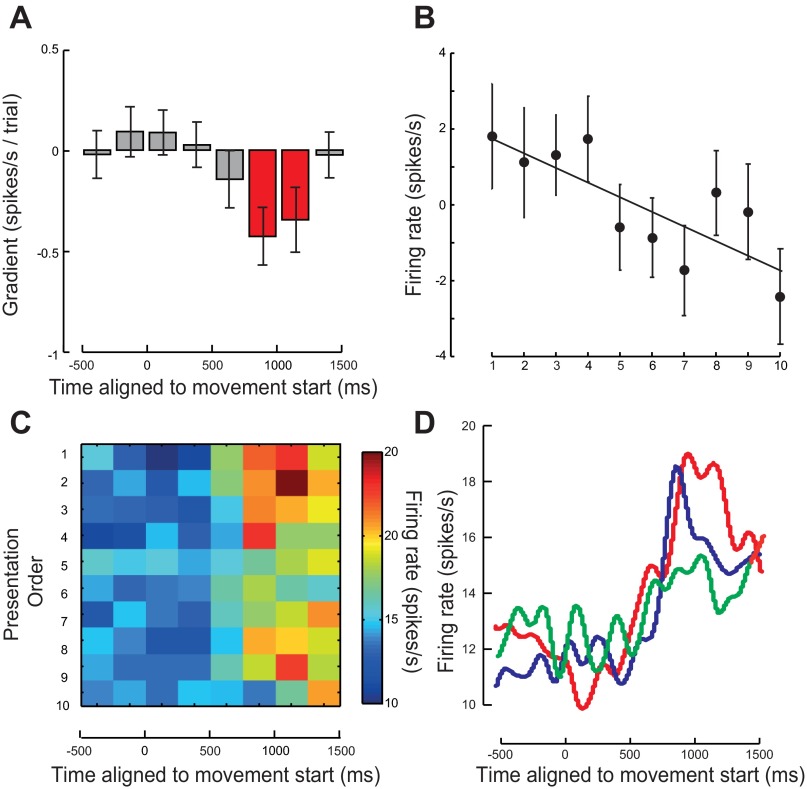
Modulation in mirror neuron firing rate when aligned to the time of observed movement onset. *A*: average rate of change of firing rate with repetition over 7–10 trials. A negative value indicates a firing rate decrease with repetition. Error bars show means ± SE. Time points where the data were significant are shown in red. *B*: average relative change in firing rate calculated from the mean firing rate averaged across mirror neurons for the 7–10 presentations of the observed action averaged for the 250 ms before the object being grasped. Error bars show means ± SE. *C*: average firing rate of all mirror neurons for each presentation. Presentation order is shown on the *y*-axis with repetitions increasing from *top* to *bottom*. *D*: average smoothed firing rate across all mirror neuron and averaged across *presentations 1* and *2* (red line), *presentations 3* and *4* (blue line), and *presentations 5* and *6* (green line).

The rate of change of firing rate with presentation for each of the 67 mirror neurons aligned to the time at which the experimenter's movement started showed a systematic and significant decrease in two 250-ms windows starting 750 ms after movement onset [*t*(66) = −3.00; *P* < 0.005: *t*(66) = −2.16; *P* < 0.05: for the significant bins; Fig. 6*A*, red bars]. This decrease in firing rate, 0.55 spikes·s^−1^·trial^−1^, equated to a decrease of 5.5 spikes/s over the 10 presentations of the same action (Fig. 6, *B*–*D*). As before, all significant linear decreases in firing rate with presentation occurred in the periods just before the object being grasped and during grasp, which occurred on average 1,090 ms after the movement onset (Fig. 6, *A*, *C*, and *D*). When aligned to movement start, this pattern of modulation was observed most clearly in mirror neurons that suppressed their firing rate either before the object grasp [facilitation mirror neurons, *t*(30) = −1.37, *P* = 0.18; suppression mirror neurons, *t*(27) = −2.42, *P* = 0.023] or subsequent to the object grasp [facilitation mirror neurons, *t*(34) = −1.5, *P* = 0.14; suppression mirror neurons, *t*(27) = −3.62, *P* = 0.001]. As before, this effect was nonsignificant if the sign of the modulation of the suppression mirror neurons was reversed before statistical testing [*t*(66) = −0.72, *P* > 0.05 and *t*(66) = 0.09, *P* > 0.05, respectively].

#### Modulation of latency of firing onset with repetition.

**Fig. 7.**
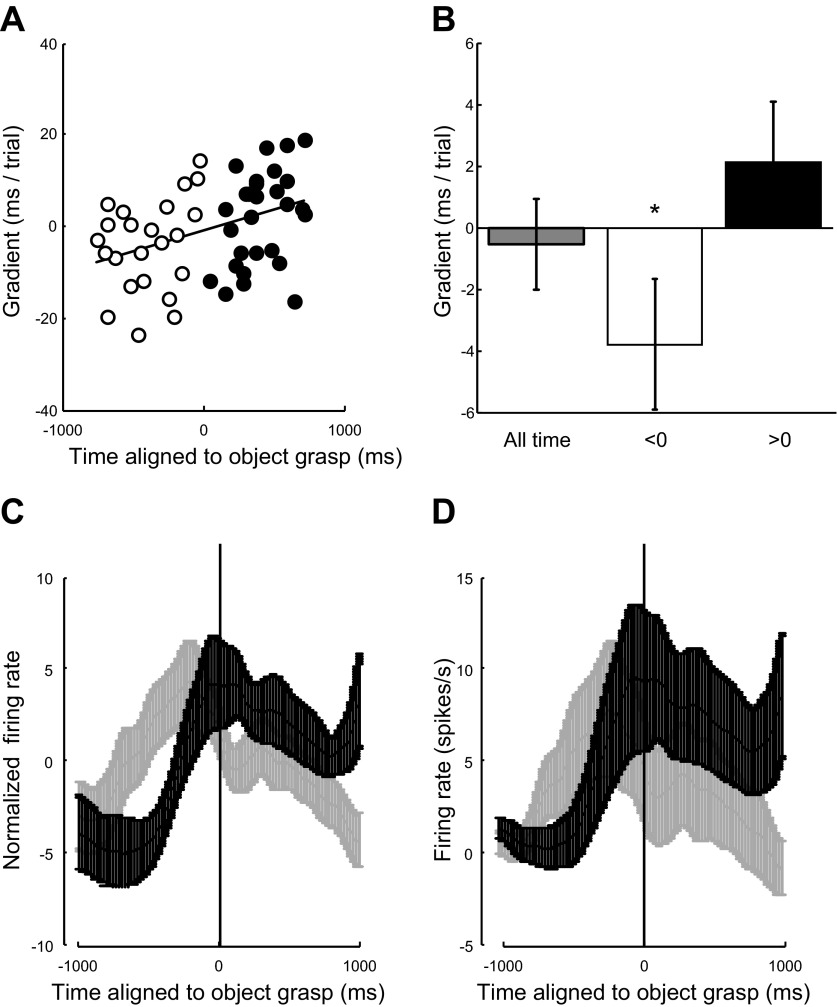
Latency of discharge in mirror neurons after beginning of the observed action. Discharge latency for all mirror neurons is centered on the time of maximal discharge. *A*: correlation of the rate of change of the latency measure across repeated trials against the time of maximum firing rate for each neuron. *t* = 0 is the time at which the object was grasped. Open circles indicate 23 neurons with maximum firing rate before (<0) the time at which the object was grasped (*t* = 0 ms) and filled circles are for those neurons (*n* = 28) that had their average maximum firing rate after (>0) the object was grasped. The solid line shows the slope of the regression illustrating the dependence between when the neuron maximally fired and the change in the latency measure across trials. *B*: average of the rate of change of the latency measure across repetitions for all neurons, those that had their firing rate before *t* = 0, <0, and those that maximally discharged after *t* = 0, >0. *P < 0.05, significant change. *C* and *D*: average firing rate for all mirror neurons that showed a decrease in latency with repetition (*n* = 26; light gray lines) and show the average firing rate for all mirror neurons that showed an increase in latency with repetition (*n* = 25; dark gray lines). Error bars show means ± SE. *C*: average of the normalized firing rate, normalized so that the mean was zero and standard deviation was equal to 1. *D*: unnormalized firing rate. The firing rates in both *C* and *D* are aligned to the time of object grasp.

The analysis of latency focused on any modulations in neuronal firing with repetition in a 1,000-ms window centered on the time that each neuron fired maximally (its center of mass) during the observation of a reach to grasp action. The analysis of 51 mirror neurons included in the analysis (see methods) revealed no significant modulation in onset latency with repetition, irrespective of whether they discharged maximally before or after the object was grasped [*t*(50) = −0.37, *P* = 0.71; Fig. 7*B*, dark gray bar]. However, there was a significant dependency between the change in latency with repetition and when in peristimulus time the neuron discharged (Fig. 7*A*; *P* < 0.005, *R*^2^ = 0.16). The results for the 23 neurons that discharged maximally before the object was grasped (Fig. 7*A*, open circles) showed a systematic decrease in the latency of their maximum firing with repetition [Fig. 7*B*, *t*(22) = −1.80, *P* < 0.05, one tailed]. In contrast, the 28 neurons that discharged maximally after the object was grasped (Fig. 7*A*, filled circles) showed no significant pattern in the modulation of latency of their maximum discharge [Fig. 7*B*, >0; *t*(21) = 1.09, *P* = 0.16]. Note that these modulations were not simply due to any trial-by-trial changes in the overall duration of each observed action, which started at the point indicated by the red dots in Fig. 1, *A* and *B*, as these were included in the regression analysis (see methods).

To investigate further whether there were any systematic differences in the firing pattern of neurons that decreased their latency of discharge with repetition, we calculated the mean firing rate for all 26 mirror neurons where there was a decrease in latency with repetition and all 25 mirror neurons where there was an increase in latency with repetition (Fig. 7, *C* and *D*, gray and black, respectively). Confirming the previous results, those neurons where there was a decrease in the latency (gray lines) on average discharged earlier and predominantly during the period before object grasp. This was in contrast to those mirror neurons where the latency increased, these neurons peaked in discharge around the time the object was grasped and continued to discharge during the observation of the action after the object had been grasped (black lines).

#### Accounting for global confounds that could explain decrease in firing rate with repetition.

**Fig. 8.**
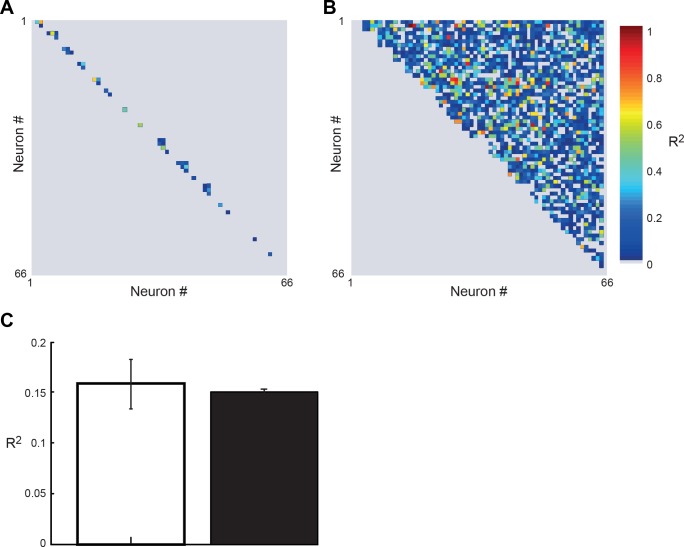
Correlations in firing rate of mirror neurons within and across different recording sessions. *A*: correlation coefficient explaining shared variance in firing rates with repetition between all mirror neuron pairs that were recorded in the same session. *B*: correlation coefficient explaining shared variance in firing rates with repetition between all mirror neuron pairs that were recorded in different sessions. *C*: average correlation coefficients for all mirror neuron pairs (*n* = 53) recorded in the same session (white bars) and all mirror neuron pairs (*n* = 2,065) recorded in separate sessions (black bars). Error bars are SE.

The previous results showed a significant modulation in mirror neuron firing rate with repeated observations of the same action. To investigate whether this decrease could be accounted for by nonspecific global confounds, for example, in attention or eye-gaze, we tested for differences in modulation in firing rate for mirror neurons that were recorded simultaneously and those recorded in different sessions. To this end we correlated the firing rate in the 500 ms before the object grasp for each trial between all combinations of pairs of the 67 mirror neurons. We then tested for differences in the correlation coefficient and gradient of the change in firing rate with repetition number between mirror neurons recorded in the same session (*n* = 53; Fig. 8*A*) and those recorded in separate sections (*n* = 2,065; Fig. 8*B*). The logic being that if the decrease in firing rate was due to a global nonspecific effect, such as a decrease in attention, this should have a global effect on all neurons recorded in the same session, i.e., identical attention, and therefore the gradient should be closer to unity and the correlation coefficient should be greater for mirror neurons recorded in the same sessions than those recorded in separate sessions. No significant differences between either the correlation coefficients calculated for pairs of mirror neurons from the same session (*n* = 53) and from different sessions [*n* = 2,065; Fig. 8*C*, white and black bars, respectively; *t*(2,116) = 0.34, *P* = 0.73] were observed.

#### Modulation of LFP power with repetition aligned to grasp.

**Fig. 9.**
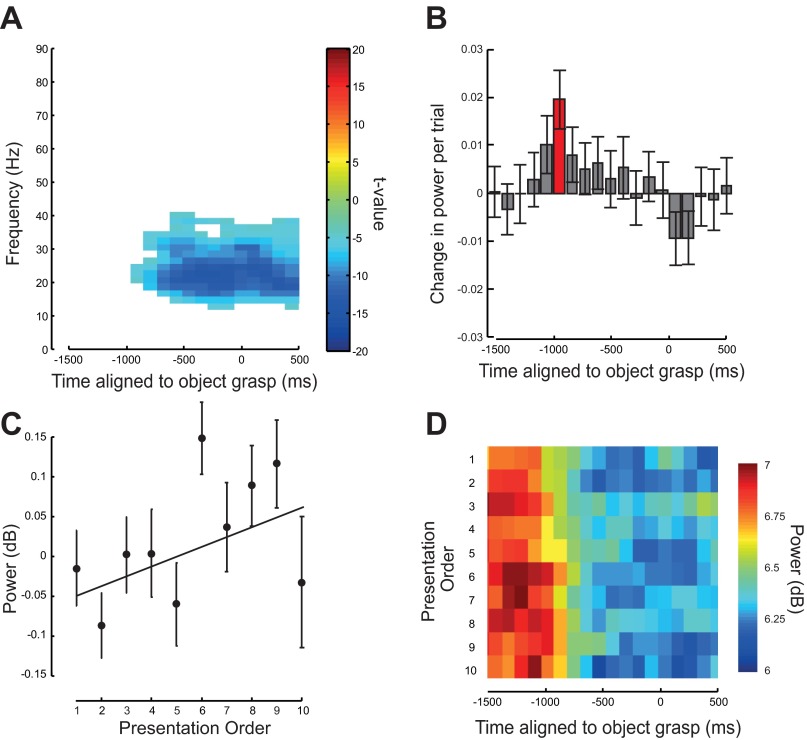
Local field potential (LFP) activity during observation of repeated actions. Analysis of F5 LFP recordings aligned to the moment the object was grasped. *A*: time-frequency SPM t-map thresholded at *P* = 0.05 family-wise error (FWE) corrected for multiple comparisons (*n* = 64 LFP recordings). The image shows where there was a significant difference in power during the period of action observation compared with the average power in a 500-ms premovement baseline window. Negative values indicate a decrease in power compared with baseline. *B*: average rate of change of power averaged over the 15- to 23-Hz frequency range with repetition. A positive value indicates an increase in power with repetition. Error bars show means ± SE. Time points where the data were significant are shown in red. *C*: average relative change in 15–23 Hz power from its mean averaged across LFP recordings for the 10 presentations of the observed action, and averaged over the 500 ms after movement onset (starting at −1,100 ms). Error bars show means ± SE. *D*: average power in the 15–23 Hz range across LFP recordings for each presentation. Presentation order is shown on the *y*-axis with repetitions increasing from *top* to *bottom*.

An initial analysis of the LFP data investigated whether there were any significant task-dependent modulation in power at any frequency between 0 and 100 Hz averaged across repetitions. Only power in the frequency range from 13 to 35 Hz showed a significant attenuation during the period of action observation starting 1,040 ms before the object was grasped (movement started on average 1,090 ms before the object was grasped), and this was significantly attenuated throughout the period of action observation (*P* < 0.05 corrected for FWE; Fig. 9, *A*, *D*, and *E*). All further analysis focused on the period where this attenuation was greatest, in the frequency range 15–23 Hz (Fig. 9*A*). The power in this frequency range showed a significant and systematic increase with repetition 1,040 ms before the moment of object grasp [*t*(63) = 3.44, *P* < 0.01; Fig. 9, *B*–*D*].

#### Modulation of LFP power with repetition aligned to movement start.

**Fig. 10.**
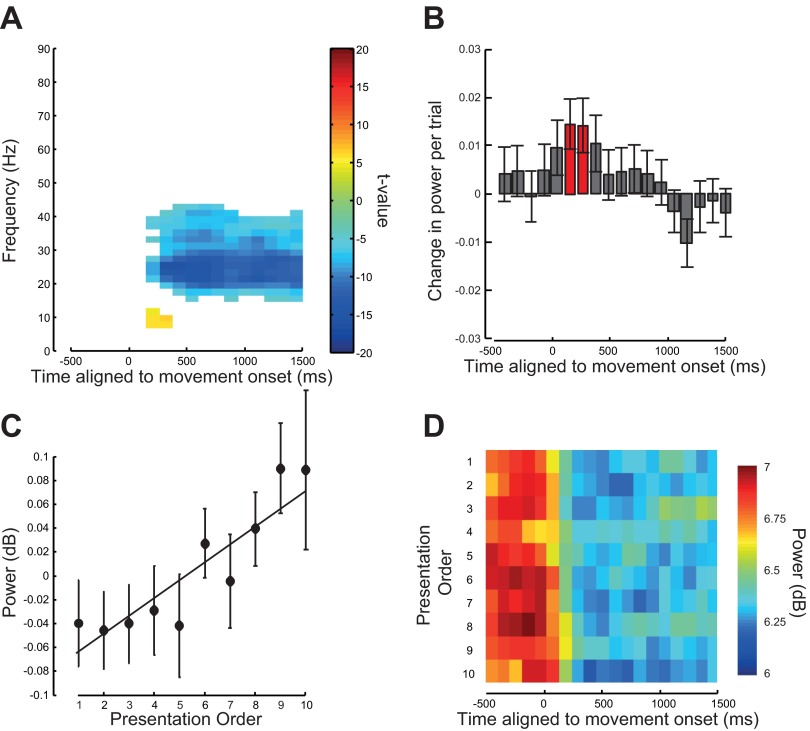
Analysis of the LFP data aligned to the moment of observed movement onset. *A*: time-frequency SPM t-map thresholded at *P* = 0.05 FWE corrected for multiple comparisons. The image shows where there was a significant difference in power during the period of action observation compared with the average power in a 500 ms premovement baseline window. Negative values indicate a decrease in power compared with baseline. *B*: average rate of change of power averaged over the 15- to 23-Hz frequency range with repetition. A positive value indicates an increase in power with repetition. Error bars show means ± SE. Time points where the data were significant are shown in red. *C*: average relative change in 15–23 Hz power from the mean 15–23 Hz power averaged across LFP recording s for the 10 presentations of the observed action averaged over the 500 ms after average movement onset (starting at 0 ms). Error bars show means ± SE. *D*: average power in the 15- to 23-Hz range across LFP recordings for each presentation. Presentation order is shown on the *y*-axis with repetitions increasing from *top* to *bottom*.

We repeated the above analysis to detect any significant task-dependent modulation in LFP power across repetitions when the data were now aligned to the onset of the observed movement. As before power in the frequency range from 13 to 35 Hz showed a significant attenuation during the period of action observation starting 150 ms after the movement started (*P* < 0.05 corrected for FWE; Fig. 10, *A*, *D*, and *E*). The power in this frequency range showed a significant and systematic increase with repetition in two time bins centered on 150 and 260 ms after movement onset [*t*(63) = 2.47, *P* < 0.05 and *t*(63) = 2.03, *P* < 0.05; Fig. 10, *B*–*D*].

**Fig. 11.**
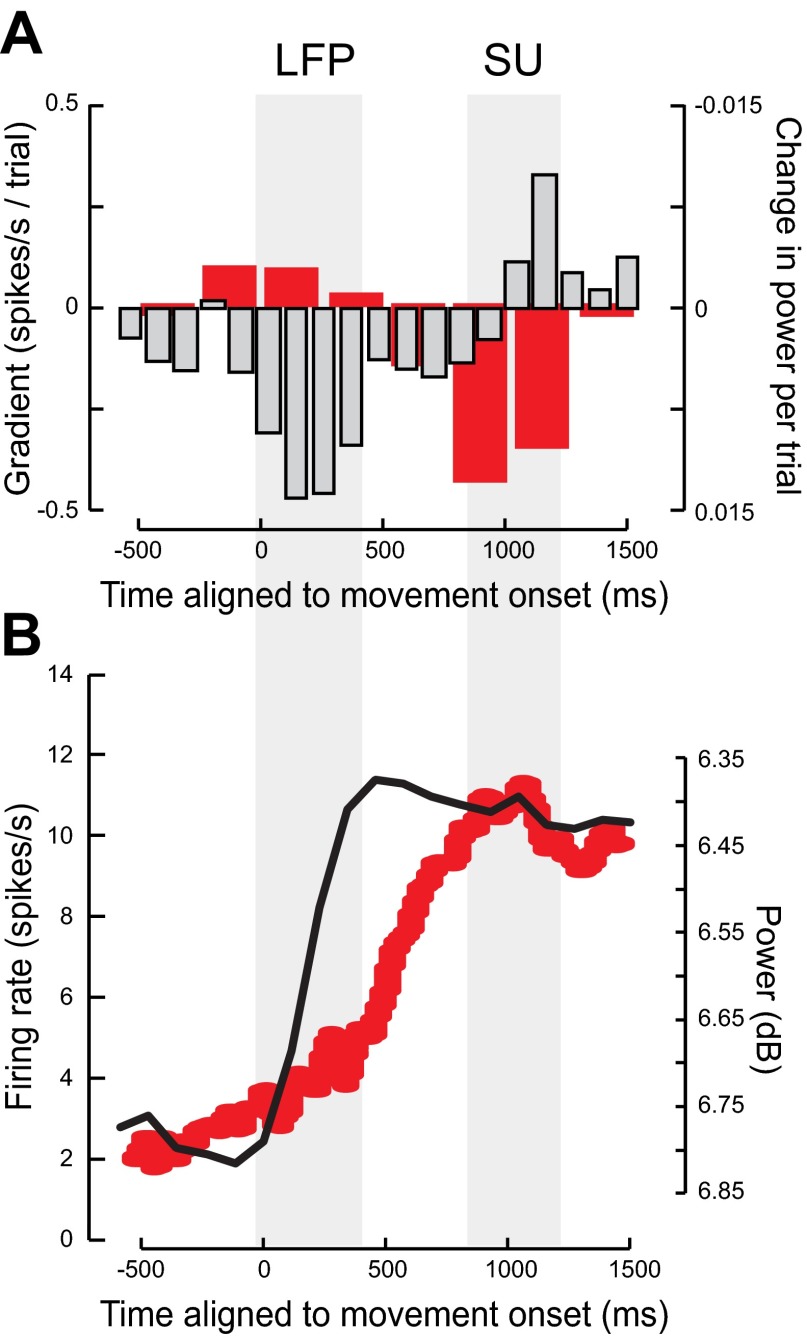
Comparison of LFP and mirror neuron firing rate data aligned to observed movement onset. *A*: average modulation in mirror neuron firing rate with repetition (red bars, *y*-axis on the *left*) and the average modulation of power in the 15- to 23-Hz band with repetition (light gray bars *y*-axis on the *right*). Note the sign of the power has been inverted for ease of comparison. The gray shaded areas show periods where there was a modulation in either the LFP power or single unit (SU) firing rate with repetition. *B*: average of the absolute difference in firing rate across all mirror neurons from a 500-ms premovement period (red line, *y*-axis on the *left*) and the average modulation in power in the 15–23 Hz compared with the average power in a 500 ms baseline window before the onset of the observed movement (black line, *y*-axis on the *right*). Note again the inversion of the LFP power scale.

A summary of the LFP and mirror neuron firing rate changes is shown in Fig. 11. On average, there was a sharp decrease in power in the 15- to 30-Hz range at the onset of the observed action (Fig. 11*B*, black line). In contrast, the mirror neuron firing rate was modulated more gradually ∼500 ms before the object was grasped and after the modulation in LFP power in the 15- to 30-Hz range (Fig. 11*B*, red line). Significant modulations in both the LFP power and single unit firing rate with repetition occurred at the points in perievent time when, on average, these two measures where maximally modulated (compare Fig. 11, *A* and *B*). In this way changes in LFP power always preceded changes in single unit firing rate.

## DISCUSSION

The aim of the current study was to test whether there were any systematic changes in neuronal activity of mirror neurons with multiple repeated observation of the same action. Here we have identified a population of 67 F5 neurons that we classify as mirror neurons because they modulated their background discharge significantly both during the monkey's own grasp of a small food reward and during observation of a precision grip performed by an experimenter seated in front of the monkey. In addition some of these neurons show response selectivity to the action observed. For these 67 mirror neurons, we show that *1*) there was a significant decrease in the firing rate of F5 mirror neurons with repetition when the monkey observed the reach and grasp phase of the observed action with the most robust changes occurring in the 250 ms before the object being grasped; *2*) in agreement with previous results (Caggiano et al. 2013), there was no significant suppression of mirror neuron firing rate from the first to the second presentation of the observed action. Instead, there was a trend of an increase in the firing rate; *3*) the latency of the onset of mirror neuron firing decreased with repetition for those mirror neurons that maximally discharge before the object being grasped; *4*) power in the 15- to 23-Hz beta range recorded in area F5 was significantly attenuated during action observation; and *5*) the power in the 15- to 23-Hz beta range was significantly increased with repetition of the observed action in the first 300 ms after movement onset.

The main finding here is that there was a significant decrease in the modulation of both firing rate and LFP with multiple repetitions of the same observed action. These results confirm and extend those of a previous study (Caggiano et al. 2013). Here we have shown that there was no significant difference in mirror neuron firing rate from the first to the second presentation as in Caggiano et al. (2013). However, here we have shown that the firing rate is systematically decreased with further repetitions. This is in distinction to the results of Caggiano et al. (2013). Here we have shown that this decrease in firing rate with repetition occurs both for facilitation and suppression mirror neurons. In other words, with repetition the facilitation mirror neurons are less active and the suppression mirror neurons are more suppressed. Indeed, if we reverse the sign of the suppression mirror neurons as in Caggiano et al. (2013) the effect with repetition was no longer significant at the population level. This suggests that the net inhibitory inputs that result in the suppression mirror neurons being suppressed during action observation are subject to a different mechanism that the presumably excitatory drives that weaken with repetition. However, further work will be required to confirm this.

There are a number of differences between this study and that of Caggiano et al. (2013). In the current study monkeys observed the same repeated action, a reach and grasp action, which was performed by the experimenter in front of the monkey. In Caggiano et al. (2013) the monkey observed videos of the reach and grasp action. There are pros and cons to both approaches. Clearly, the most obvious difference is that when employing videos as the stimulus one can present precisely the same stimulus with every repetition, whereas when observing the experimenter there would be trial-to-trial variance in the precise kinematics of the observed action. Therefore, there are some differences in the reproducibility of the observed action between this study and that of Caggiano et al. (2013). However, one of the important characteristics of mirror neurons is that they have been shown to discharge to action categories (Gallese et al. 1996); therefore, the minor trial-to-trial variations in the experimenter's performance should not have a measurable or systematic effect on the mirror neuron selectivity. In addition, using videos to control for the visual stimulus comes with a cost. It is known that only a subset of mirror neurons that respond to live actions respond to videos of the same action (Caggiano et al. 2011), reducing the potential sample of mirror neurons.

### 

#### Global changes in behavior do not explain the adaptation in mirror neuron firing rate.

One possible, perhaps trivial, explanation for the results presented here would be that the monkey was paying less and less attention on each successive trial. Although further studies will be required to address this question, one major argument against this is that the modulation was only found for a specific time period of the observed action, i.e., just before the object being grasped. If the modulation found during repeated trials was simply due to a decreasing level of attention with increasing repetition number, then one could expect the modulations to occur throughout the entire time window. Secondly, there was no significant difference when comparing the correlation of the firing rate modulation with repetition between mirror neurons that were recorded in the same session and those recorded in different sessions (Fig. 8). If the decreases in firing rate with repetition were caused by some global nonspecific changes, such as attention, then the amount of shared trial-to-trial variance would be expected to be larger for mirror neurons recorded simultaneously. This was not observed. Finally, here we showed that the latency of mirror neuron discharge before object grasp significantly decreased with repetition. If the decrease in firing rate with repetition reported here was due to a decrease in the level of attention with repetition, then the latency of firing rate discharge would be expected to increase with repetition and not decrease. Taken together the data presented here would be consistent with the suppression we observed being driven by the repeated presentation of the same stimulus and not by either a general attention effect or by a reward-related activity.

One other possible explanation of the results presented here is that the monkeys spent less time looking at the action with repeated observations. In this experiment eye movements were not recorded so we cannot directly address this concern. However, there are a number of pieces of evidence that would suggest that looking time is not a sufficient explanation of the results. Firstly, as described above there is no evidence of a global nonspecific effect, such as looking time, that could explain the results (see also Maranesi et al. 2013). Secondly, a decrease in looking time cannot explain the significant decrease in the latency of mirror neuron discharge with repetition observed. Finally, in a separate experiment that did record eye gaze no evidence was found that the monkeys significantly decreased their looking time during repeated trials of action observation and spent a high proportion of their gaze time looking at the actions (Philipp et al. 2013).

#### Models of repetition suppression.

There are three main models that have been proposed to explain repetition suppression effects: the fatigue model, the sharpening model, and the facilitation model. Of these both the fatigue model and the sharpening model are proposed to be driven by bottom-up mechanisms, being dependent on the visual properties of the stimulus (De Baene and Vogels 2010; Summerfield et al. 2011). The fatigue model proposes that repetition suppression occurs as neurons tuned to the visual properties of the stimulus are less likely to discharge when the same stimulus is presented in quick succession, either due to the hyperpolarization of the neurons after discharge to the first presentation and/or due to a decrease in presynaptic transmitter release (cf. Grill-Spector et al. 2006). The sharpening model proposes that with successive repetitions the tuning curve of the stimulus across the population of neurons is tightened such that only a subpopulation of the neurons will decrease their firing rate.

These two models are typically invoked to explain repetition suppression effects. Neither model could account for the modulations in firing rate observed here and by Caggiano et al. (2013), where there was a trend for an increase in firing rate, albeit nonsignificant, from first to second presentation. In the facilitation model the modulation in neuronal response with repetition reflects changes in the synaptic potentiation between neurons, enabling a faster and more efficient processing of the stimulus leading to a modulation in the latency, duration, and firing rate of each neuron discharge with repetition. In distinction to the sharpening and fatigue models, the facilitation model depends on top-down modulation and it has been proposed that within this framework, repetition suppression and even augmentation can be partially explained by the expectation of the forthcoming stimulus (de Gardelle et al. 2013; Summerfield et al. 2008; Garrido et al. 2008, 2009). This framework can also explain the results observed here; monkeys were only unable to predict the observed action on first trial of a blocked series of trials. Having seen the first trial, they knew that all subsequent trials would be performed with the same action. This predictability of the observed action could have a profound effect on mirror neuron firing rate, as it would modulate the precision of the predictive model (Kilner et al. 2007; Friston et al. 2011). Therefore, the change in firing rate that we report here may reflect changes in the subjects' confidence in the prediction of the observed action as a function of repetition.

#### Significance for repetition suppression studies using fMRI.

It has previously been proposed that fMRI adaptation techniques could be employed as an indicator of the presence of populations of mirror neurons in humans (Dinstein et al. 2007, 2008). However, the results of studies employing this technique to investigate the presence of mirror neurons in humans have had mixed results (Dinstein et al. 2007; Lignau et al. 2009; Chong et al. 2008; Kilner et al. 2009; Press et al. 2012) leading some to speculate that mirror neurons themselves do not modulate their firing rate with repetition of the observed action. Here we have demonstrated that mirror neurons in area F5 show small yet significant and reproducible modulations in their firing rate, their latency of firing, and the amplitude in beta power recorded in the LFP with multiple repetitions of a natural grasping action. Although these results show for the first time that mirror neurons adapt over multiple repetitions of the same observed action, and are consistent with the studies that have reported significant fMRI adaptation when observing the same, repeated action, the results presented here should not be interpreted as proof that fMRI adaptation can be used to identify mirror neurons in humans. There are a number of important differences between the analysis here and those employed in fMRI adaptation. Previous fMRI studies have investigated changes in blood oxygen level-dependent (BOLD) signal between only two presentations of the same stimulus (Dinstein et al. 2007; Chong et al. 2008; Lignau et al. 2009; Kilner et al. 2009; Press et al. 2012) whereas here the effects were observed over 7–10 repetitions of the same action. Here we found no evidence in favor of a significant change in mirror neuron firing rate from the first to the second presentation, neither a facilitation nor attenuation in firing rate. The results of the current study suggest that any changes in BOLD signal due to fMRI adaptation between the first and second presentation of the same observed action would at best be small and may well not be significant at all. This is precisely what has been found. (Dinstein et al. 2007; Chong et al. 2008; Lignau et al. 2009; Kilner et al. 2009; Press et al. 2012). Given this, it would clearly be of great interest to test whether the fMRI adaptation effects are greater given multiple repeats of the same observed action as opposed to just one repeat. The results of this study would predict that the fMRI adaptation effect would indeed be greater with multiple repetitions. Finally, the original logic of using fMRI adaptation was to investigate adaptation between the same action when observed and then executed or vice versa, so called cross modal repetition suppression. It is important to highlight that here we have shown that mirror neurons adapt when monkeys repeatedly observed the same action and that the results should not be interpreted as evidence that mirror neurons show cross modal repetition suppression. Although this is an important and necessary step in understanding the modulations of mirror neurons discharge with repetition, it remains to be seen whether mirror neuron firing rate will be modulated in the same way between observation and execution conditions or even when observing different classes of observed actions.

#### LFP activity during action observation.

Here we have shown that power in the beta band of the LFP, a measure reflecting summed local synaptic activity, was augmented with repetition. This augmentation occurred before any modulation in the firing rate (Fig. 11*A*). Whereas the systematic decrease in firing rate occurred in the 300 ms before object grasp (Fig. 11*A*, dark gray bars), the systematic changes in the beta power occurred during the first 400 ms after movement onset (Fig. 11*A*, light gray bars), that is, a further 400 ms before any changes to the firing rate. This same pattern of modulation with repetition was also found in the time course of LFP beta power and firing rate changes during action observation (Fig. 11*B*, black and red lines). At observation of movement onset, the LFP beta power was almost immediately attenuated (Fig. 11*B*, black line) reaching a minimum ∼450 ms after observed movement onset. In contrast the firing rate of mirror neurons only really began to change after the LFP beta power had been fully attenuated (Fig. 11*B*, red line) and reached a maximum change in firing rate from baseline at the time the object was grasped ∼1090 ms after movement onset. We have previously reported a reciprocal relationship between the selectivity of beta power and F5 neuronal discharge during different types of grasp (Spinks et al. 2008).

Previous human neuroimaging studies employing EEG or MEG have demonstrated an attenuation of cortical oscillatory activity during periods of movement observation that is similar to that observed during movement execution in the 15- to 30-Hz (beta) range (Hari et al. 1998; Cochin et al. 1998, 1999; Babiloni et al. 2002; Kilner et al. 2009; Press et al. 2010). The attenuation of the beta oscillations during action observation has been interpreted as evidence of a mirror neuron system in humans (cf. Rizzolatti and Craighero 2004). Although it is well established that this synchronous oscillatory activity in the beta range principally originates in the primary motor cortex (M1; Murthy and Fetz 1992; Hari and Salmelin 1997), it has been argued that given the dense anatomical connections between inferior frontal gyrus and M1 (Matelli et al. 1986; Dum and Strick 2005; see Davare et al. 2011), it is likely that M1 receives increased postsynaptic activation during periods of action observation. Here we show for the first time that oscillatory activity in the beta range recorded from area F5 is significantly attenuated during action observation. Although further work will be required to investigate the functional relationship between modulations in oscillatory activity in M1 and F5 during action observation, the finding that this activity in F5 is modulated during action observation strengthens the argument that modulations observed in human beta-oscillatory activity during action observation does indeed reflect activity in connected areas of cortex known to contain mirror neurons.

#### Conclusions.

Previous studies have argued that the observation of a significant fMRI adaptation effect is evidence for the existence of mirror neurons in humans (Kilner et al. 2009). Here we have shown that F5 mirror neurons of the macaque monkey significantly modulate their pattern of firing on multiple repetitions of an observed action. With repetition we have shown that mirror neurons decrease their firing rate and decrease their latency of discharge and that the power in the beta-frequency range in the LFP recordings from area F5 is augmented with multiple repetition. Although the neuronal changes underlying the BOLD response are not fully understood, it is thought that the BOLD response is likely to reflect both changes in firing rate and synaptic activity (Logothetis 2008; Rosa et al. 2011; Attwell et al. 2010; Harris et al. 2011). The repetition-related modulations in single neuron firing rate and synaptic activity that we report here are subtle. Whether these changes, reproduced over a large population of many mirror neurons, could lead, at the neurovascular level, to the decrease in the BOLD signal revealed using fMRI adaptation remains a question for future studies.

## GRANTS

This work was funded by the Wellcome Trust, UK.

## DISCLOSURES

No conflicts of interest, financial or otherwise, are declared by the author(s).

## AUTHOR CONTRIBUTIONS

Author contributions: J.M.K. and A.K. analyzed data; J.M.K., A.K., and R.N.L. interpreted results of experiments; J.M.K. prepared figures; J.M.K., A.K., and R.N.L. drafted manuscript; J.M.K., A.K., and R.N.L. edited and revised manuscript; J.M.K., A.K., and R.N.L. approved final version of manuscript; A.K. and R.N.L. conception and design of research; A.K. and R.N.L. performed experiments.
